# Identifying Trustworthy Experts: How Do Policymakers Find and Assess Public Health Researchers Worth Consulting or Collaborating With?

**DOI:** 10.1371/journal.pone.0032665

**Published:** 2012-03-05

**Authors:** Abby S. Haynes, Gemma E. Derrick, Sally Redman, Wayne D. Hall, James A. Gillespie, Simon Chapman, Heidi Sturk

**Affiliations:** 1 Sydney School of Public Health, University of Sydney, Camperdown, New South Wales, Australia; 2 The Sax Institute, Haymarket, New South Wales, Australia; 3 Institute of Public Goods and Policies, Consejo Superior de Investigaciones Cientificas, Madrid, Spain; 4 University of Queensland Centre for Clinical Research, University of Queensland, Herston, Queensland, Australia; 5 Menzies Centre for Health Policy, University of Sydney, Camperdown, New South Wales, Australia; 6 School of Medicine, University of Queensland, Herston, Queensland, Australia; Johns Hopkins Bloomberg School of Public Health, United States of America

## Abstract

This paper reports data from semi-structured interviews on how 26 Australian civil servants, ministers and ministerial advisors find and evaluate researchers with whom they wish to consult or collaborate. Policymakers valued researchers who had credibility across the three attributes seen as contributing to trustworthiness: *competence* (an exemplary academic reputation complemented by pragmatism, understanding of government processes, and effective collaboration and communication skills); *integrity* (independence, “authenticity”, and faithful reporting of research); and *benevolence* (commitment to the policy reform agenda). The emphases given to these assessment criteria appeared to be shaped in part by policymakers' roles and the type and phase of policy development in which they were engaged. Policymakers are encouraged to reassess their methods for engaging researchers and to maximise information flow and support in these relationships. Researchers who wish to influence policy are advised to develop relationships across the policy community, but also to engage in other complementary strategies for promoting research-informed policy, including the strategic use of mass media.

## Introduction

Journal articles, reports and government briefs are not the only sources of research used in policy development. Policymakers often prefer face-to-face consultations with researchers, or ‘experts’, in lieu of reading research [1], [2], particularly when policy must be formulated under tight time constraints. Policymakers also benefit from researchers serving on government committees and advisory groups, contributing to public and stakeholder forums, conducting research in partnership with policymakers, and disseminating relevant research through strategically developed policy networks [3]–[5]. Good interpersonal relationships between researchers and policymakers are consistently identified as key facilitators of research-informed policy development [6]–[9], yet little is known about how these relationships are formed or about the ways that policymakers identify researchers whom they invite into the ‘inner circle’ of policy development. We know that policymakers' research requirements differ according to the type of policy on which they are working and the phase of its development [10]. Similarly, their use of researchers varies in response to changing policy contexts [2]. This suggests that different researcher characteristics will be sought for different policy activities. Knowing if policymakers use the same criteria to select researchers for exploratory agenda-setting dialogue as they do for supporting legislation-focused consensus building or for evaluating interventions would enable researchers to use their expertise more effectively to pursue their goals of influencing policy.

Trust is considered to be a critical factor in researcher-policymaker relationships [7], [11], [12], and a facilitator of research utilisation [13]. Trustworthiness, comprising *competence/ability*, *benevolence*, and *integrity* [14], [15], reduces uncertainty and perceptions of risk in relationships where one party is relying on another [13]. Policymakers, for example, rely on researchers when they act on their advice; when they use them in the public sphere; and when they bring them into confidential behind-the-scenes deliberations [2]. Yet we know little about how policymakers assess researchers' trustworthiness.

Empirical studies have shown relationship-based strategies to have some effect on the use of research in policy making [7], [16]–[18], but our understanding of activities that are independent of researcher-policymaker relationships is less clear. Many researchers appear in the media as commentators and advocates [19] in order to inform the general public and influence policymakers—politicians in particular—who are known to be “dedicated media-watchers” [20], but there is scant information about how researchers' media roles affect policymakers' awareness of and response to them or their work.

We previously conducted an interview-based study of relationships between policymakers and researchers [2], [4]. We found that researchers were used to galvanise policymakers' thinking, to clarify research and provide independent advice, to persuade colleagues and stakeholders, and to defend research-informed policy [2]. Here, we expand on those findings by presenting novel data from the interviews describing how research-engaged Australian civil servants, parliamentary ministers and ministerial advisors identify and assess researchers with whom to consult (or avoid), and how different policy activities affect these processes. We reflect on the critical role of trustworthiness, and comment on the implications for policymakers, and for researchers who wish to have more policy influence.

This paper focuses on explicating policymakers' self-reported views and behaviours regarding their selection of researchers. For more theoretical analyses of the relationship between researchers and policymakers in this study, see Haynes at al. 2011a [4] (which considers strategies that researchers use to influence policy) and Haynes et al. 2011b [2] (which focuses on the ways in which policymakers use researchers).

## Methods

Policymaker participants were identified from policy case examples described by researchers interviewed in a previous study. This earlier study comprised a national survey of Australian public health researchers [21], followed by semi-structured interviews with 36 researchers who had been peer nominated in the survey as ‘most influential’ [4]. In interviews, researchers were asked (a) to nominate examples of public health legislation in the past five years where they had personally observed policymakers utilise research and/or researcher advice, and (b) to name the policymakers (civil servants, ministers and ministerial advisors) concerned. These policymakers were contacted and invited for interview, during which other significant players were also identified (see table 1).

**Table 1:** Categories of study invitees and participants.

Role*		Invited	Participated
1	Civil servant	20	18
2	Ex-premier, minister or ex-minister	8	4
3	Ministerial advisor	5	4
4	Non-government organisation officer	5	4
5	Other (community group representative, independent advocate)	2	2
Total		40	32

*Only categories 1–3 are reported on in this paper.

The civil servants were middle- to high-ranking staff in Departments of Health and health related regulatory or central agencies in the two most populous Australian states: New South Wales and Victoria. All were engaged in state-wide policy formation including the development and implementation of legislation, mandatory guidelines, and intervention programs. Seven of the 18 had roles with briefs to foster innovation and inform policy by collating or commissioning research. Six had or were undertaking PhDs and three had extensive clinical experience. The ministers and advisors were currently or previously associated with Health portfolios and/or Premierships (heads of government) in NSW or Victoria. One minister had clinical experience and two of the advisors had worked as civil servants.

Semi-structured interviews with these participants addressed broad questions about the relationship between research and policy. The questions about how researchers were identified and assessed were approached from three angles. First, we asked, how did the interviewees came to work with the researchers involved in the policy example under consideration, and what was their assessment of the researchers' contribution? Second, how did the policymakers and their colleagues identify and assess researchers in the course of their day-to-day work (that is, if they ever sought researchers)? Third, if the policymakers were required to consult with a researcher in a field that was new to them, how would they go about finding that person and assessing their suitability? This combination of generalised and specific questioning, together with “tracing” from a known policy example [22], was intended to capture a spectrum of behaviours and minimise idealised accounts of the process.

The first phase of coding used simple categories and subcategories stemming from the interview questions. How did policymakers:

1. Identify researchers/experts: (a) in general, (b) in new circumstances, and (c) in relation to the policy example?
2. Assess researchers/experts: (a) in general, (b) in new circumstances, and (c) in relation to the policy example?

Each category was then subcategorised to accommodate for differences in attitudes and behaviours between civil servants (employees in health-related government departments) and politicians (ministers and their advisors). The data were divided in this manner during coding because the research needs and behaviour of civil servants and politicians differed in important ways. Although ministers and advisors also have distinct roles and responsibilities their *research need*s *and goals* were considered sufficiently similar to be grouped together in this paper. Any differences are described. A second phase of coding explored the data for themes relating to the strategies that researchers in our earlier study reported using to increase policymakers' awareness of research and uptake of research-based advice [4].

The study was approved by the Behavioural and Social Sciences Ethical Review Committee of the University of Queensland, protocol number 2009000340. Informed written consent was obtained from all participants involved in the study. All participants have been de-identified.

## Results

### Civil servants' identification of researchers

Civil servants in research-related roles (nearly two fifths of the civil servant interviewees) were confident that they had sufficient knowledge of Australia-based and international researchers to be able to identify experts with whom to engage:

*We actually do approach researchers because we have a high skilled policy team across a number of areas who are always reading, doing literature reviews, doing evidence reviews and so on….So we'll start making our own judgements about people that we want to speak to, and we'll engage them.*

The remaining three fifths who did not have research-specific roles were less confident, but they felt able to identify a few leading Australian researchers from their familiarity with *“key papers”* and via their personal or organisational contacts with researchers.

When asked how they did or would identify new researchers, over four fifths of civil servants said the primary route was through their networks. Foremost in these networks were departmental colleagues and policymakers in other jurisdictions: *“The number one strategy would be using our policy colleagues to see if they have any recommendations”*. Other network members were researchers and other *“experts”* already known to the department within academia, NGOs, knowledge brokerage organisations, senior health service management or professional associations: *“It's like pyramid selling. You have a researcher who's very key and understands policy interaction but that person also has their own networks and so they bring with them the wealth of their own networks, not just themselves.”*

A third of civil servants used conferences and research forums: “*Whenever I go to a meeting or to an annual forum or to a conference…you're on the lookout for people who have got an interesting perspective”.* Two departmental branches engaged researchers primarily through open tendering processes, but the civil servants frequently encouraged researchers with whom they had already worked to apply. Researchers were also known to self-identify: *“they contact us and ask why aren't they on a committee”* or *“they come to us…through another mechanism [for example] it's on the back of some research they've done and then that opens the door”.*

Senior civil servants took advice from the Chief Health Officer, their director general or minister about where to obtain expertise and who to include on taskforces, working parties and committees. Scanning names from previous committees and taskforces was another option. A civil servant explained that she could establish a committee membership within a few days using this method.

Methods for identifying researchers appeared ad hoc, and there was a sense that this process differed not only between departments and branches, but also from person to person. None of the interviewees mentioned a formal procedure and one fifth believed that this was problematic: *“I don't think it's a very good systematic way to get the best for both sides.”* This concern was based on the desire for a spectrum of views: “*Diversity is important – everyone has an opinion and it can get fixed. It's good to hear different opinions, to get the breadth of an issue and consider the different ways of seeing it.”* Yet the pool of researchers was considered to be stagnant in several branches, including branches that used open tendering processes:

*… often it's the same old researchers, and I think that's detrimental to the research community because it's the same message…. You constantly see the same people sitting on committees at whatever level they are, and I'm quite sure they're not the only people who are the experts in that field.*

This accords with Ritter (2009) [1] who found a similar use of “the same small group” of researchers by Australian policymakers engaged in drug policy development.

Representative diversity may have also been limited by the noticeable tendency to work with researchers in the same locality: *“It's just the way it generally happens…we also do engage with people outside of X… but naturally you tend to deal with the people that are in proximity with you.”* Interviewees observed this tendency in other departments too:

*you do tend to go to the same people quite often and you'll find that each jurisdiction will have their favourite people…. Our colleagues in [an Australian state] had their people, and [another state] had their people, so you are a bit narrow in terms of your own state quite often.*

Despite acknowledging the importance of personal connections, some interviewees cautioned against over-emphasising their influence. One-to-one relationships increased dialogue, but no matter how effective or how senior the connections were they were not a panacea for advancing research-informed policy: the nature of the bureaucratic machine involving multiple branches, departments and strata of government meant that policy proposals had to be deemed acceptable by an aggregation. For example, when asked if a single researcher-advocate could leverage their relationships to affect departmental decision-making, a civil servant told us:

*Not really. I mean if it was a small piece of work. But in large scale public policy it's just not going to happen because there are so many gates along the way for it to be screened out, where that particular advocate won't have a level of influence whatsoever.*

### Politicians' identification of researchers

Politicians tended to see researchers as a subgroup in the pool of *“experts”.* They made little distinction between experts who were prominent clinicians, senior health services managers, leaders of professional associations, NGO executives or university professors.

Like civil servants, politicians found researchers through their professional networks but primarily via departmental staff and their immediate colleagues—advisors and other ministers: *“My chief of staff met somebody who told them about somebody who said look at X and Y's work…”*. Six of the eight commented that external contacts in NGOs, academia and stakeholder groups were also important starting points for building a network of expertise: *“… it's the ripple effect. One person tells you about another person, that person tells you about another couple of people and so it's an arithmetic progression”.*

Unlike civil servants, three quarters of the politicians relied on media profile as a means of identifying researchers: *“We approached X because she was recognised by the media as a top authority”*. In some cases, media presence alone was considered to be commensurate with expertise: *“… the media is used as a proxy for being an expert in the area. So a high media profile is worth more than 10 pages of publications”.* For one advisor, it was the only means of identifying experts: *“I have absolutely no idea how I would go about identifying someone if there wasn't an obvious expert prominent in the media”.*

Politicians talked about being aware of researchers with high profile reputations for contributing to policy: *“There are some researchers who have in the past had played important roles in the structure of the current health system. People like X helped create [a national health administration scheme]. Their reputation precedes them”.* A virtuous circle of influence was evident for this elite group of well-known, policy-effective researchers: *“We just knew X was a public health guru, so I think it was just probably the fact that he was known that we sought him out for his opinion…”.* It was not possible to disentangle the route of identification for these experts; they were very well known at departmental level and may have been brought to politicians' notice through that route, but their association with significant policy reform, or their high media profile, may have endowed them with an independent *“stellar reputation”*: *“I think you know them yourself. You read about them. You see them somewhere else or somebody tells you about them.”*

Like their departmental colleagues, politicians also reported being ‘cold canvassed’ by researchers on occasion. But when these researchers secured a face-to-face meeting, their credibility was assessed by the civil servants and/or advisors who provided a background briefing. Direct contact between ministers and experts was seen by one civil servant as a frustrating complication: it required retrospective planning, second-guessing the researcher's agenda or chasing the researcher in order to provide a meaningful brief. It was also perceived as a missed opportunity to utilise departmental expertise:

*Quite often you'll find people think that “Well if I've got in the Minister's ear I'm fine…”. It's actually more irritating to have a public health researcher who's gone straight to the Minister rather than via an advisor. Extremely frustrating. Because what happens is if they're lucky enough to have got an appointment with the Minister we will have had to brief the Minister about what we think this person's going to talk about… It's much better to strategise with the department about how you use that opportunity.*

Two advisors said that they avoided unsolicited meetings with researchers. One suggested that without a personal recommendation or knowledge of the researcher, there would be no basis for discussion:

*If somebody who I had never heard of from a university called me up to discuss an issue that was within my remit as an advisor, I wouldn't know how to handle that…. I mean if I'd never heard of them I wouldn't know “Who are you? What standing do you have? Do I trust your advice?”*

### Assessing researchers

There was a high level of congruence between civil servants and politicians about the factors they considered when deciding which researchers to work with, but these factors were weighted differently depended on the policymaker's role, and the phase and type of policy development in which they were engaged.

### Trustworthiness

Trustworthiness was considered to be an essential attribute by nearly all policymakers, shaping how researchers were sought and assessed. The conceptualisation of trust appeared to incorporate many of the attributes detailed below.

### Reputation

Policymakers naturally sought researchers in whom they would have confidence, but the basis of this confidence differed between politicians and civil servants. Politicians, in particular, preferred *“reputable researchers who belong to a reputable organisation”, as* indicated by impressive academic status and, to a lesser extent, affiliation with prestigious universities or institutes: *“We were confident because he had…his title, and lots of letters after his name”*. Recommendations from trusted colleagues, network contacts, and other researchers that indicated a researcher was held in high esteem within the academic community were also powerful: *“We know we can trust his opinion because he's well respected in his field”*. Academic status was particularly important when researchers were sought for a public role because: *“Convincing the general public, convincing the media, often relies on having an expert from a sandstone university”*. A researcher's credibility could also ‘rub off’ on the minister and their proposal: *“[I want researchers who are] well regarded by their peers so…that when you quote your source people are going, ‘Oh yes, I know that person and they're credible’. So you're using their reflected competence.”*

High profile academic credentials appeared less important to civil servants, many of whom assessed researchers on their body of academic work, the policy-relevance of their track record and/or on personal experience of working with them. Civil servants often used researchers for highly specific tasks that occurred behind-the-scenes, so a researcher's expertise in a particular method of intervention evaluation, say, or the niche specialism of a research institute were more valuable than their academic status. As one civil servant pointed out, most policy-effective researchers are *“committed to outcomes and making a difference…so they're not always the most lauded professors and academics”*. Also, in the case of commissioned research, the ability of researchers and agencies to deliver reasonably costed quality work within an agreed timeframe was paramount: *“If you believe that what they've delivered in the past in a timely, accurate, professional and scientific way is good, then you'll tend to go back to that researcher or research agency”*.

### Independence

All policymakers valued researchers' independence. It not only enabled researchers to provide the *“frank and fearless advice”* that policymakers say they sought; it also delivered a political pay-off in that policy was seen to be guided by ‘objective’ science rather than political expediency: *“We need independent advice that can also be perceived as independent advice”.* Public perception of researchers' independence was mentioned as a consideration by more politicians (6/8) than civil servants (4/18). (See Haynes et al. 2011 [2] for a more detailed discussion of independence and political use.)

### Pragmatism

Researchers were highly valued when they were able to move away from *“pure”* research and engage with the *“messy real world”* using *“flexible”, “non-dogmatic”* and *“problem-solving”* approaches. This assessment was underpinned by policymakers' preference for research which is ‘fit-for-purpose’ rather than ‘elegant’. However, there was speculation that most researchers, including some with established policy-related research track records, were unequipped methodologically and temperamentally to cope with the complexities that applied research demanded. One civil servant explained how he had sought help from a government-funded researcher:

*[I said] “You've got to help me with this, I feel out of my depth.” [The researcher] actually came up with a framework that was so pathetic we couldn't use it. It was just not relevant. It wasn't practical. I remember being so frustrated: ‘How do we get people to evaluate this stuff?’ when they [researchers] didn't really want to go there. It felt to me like it was all just a bit too hard.*

### A “helicopter perspective”

Over half the interviewees said they valued researchers with an authoritative breadth of knowledge who could *“cut across the issues”*. This was particularly true in agenda-setting and early policy development when politicians were wrestling with ideas for *“bold”* reform and civil servants were weighing up alternative strategies for ministerial consideration. The researcher's own studies might focus on a specialist subfield which was particularly valuable to civil servants for designing intervention programs or crafting the fine details of policy, but their ability to situate advice within the ‘big picture’ and have a broad body of knowledge at their fingertips made them an invaluable resource for consultation: *“In order to engage government you've got to be able to show that you encompass research.”* Such researchers were treated as representatives of science, thus a single researcher could become *“our expert voice”*.

### Understanding government: the need for balance

Cultural differences between academe and government were mentioned by almost half the policymakers. Although several talked about the positive aspects of academe, all the politicians and two thirds of the civil servants preferred to work with researchers who had a solid understanding of government that included a knowledge of public health infrastructure, bureaucracy and parliamentary processes, and the socio-political history of policy reform: *“the most important thing in a good public health researcher in my view is that they have a very good understanding of politics, or what makes policy change, and know what the triggers are of pushing change.”* These researchers, they explained, appreciated the incremental nature of policy development, and the necessity for compromise and *“bringing people along with you”*. This meant that their advice was more *“balanced”* and *“realistic”.*

Having a *“balanced approach”* was particularly important when it came to policy commentary. Half the politicians and a third of civil servants disparaged researchers who *“came out with guns blazing”*, especially when they made *“wild claims or wild predictions”* that drove policy in directions that were seen as neither constructive nor evidence-informed.

A third of policymakers described soured relationships with researchers that they attributed to researchers' *“unrealistic expectations”* about government process, and a failure to appreciate the *“big, complex picture in which we work”.* They argued that these researchers patronised policymakers, felt *“disillusioned”, “threw rocks”* and failed to respect confidentiality about behind-the-scenes conversations. These experiences made many policymakers keen to avoid researchers who were not known to be policy-savvy. The acknowledged low level of research utilisation in one ministerial office was attributed to this researcher-policymaker disjuncture: *“It's uncomfortable for us to seek research out because the people who'll provide that research aren't familiar with our processes”*. This had contributed to a workplace culture in which researchers were seen as extraneous:

*“We just set up a group looking at X* [a contentious social issue with significant associated research]*…, the minister asked me to set that up and I did, and I didn't even think of seeking out a researcher. I guess that's a good example of, in practice, us not even thinking of academics as relevant.”*

### Collaborative skills

The cross-cultural challenges of collaboration were minimised when researchers were *“good team players”*; *“thoughtful, open to conversation and dialogue”.* Collaboration was facilitated by researchers making themselves available when needed—sometimes at very short notice; being friendly and easy-going; and being constructive in their criticism: *“It's easy to be a critic but it's often harder to be helpful”*. Contracted partnerships also required that researchers were “*flexible with their research design”* in response to *“real world”* demands.

All policymakers appreciated researchers with these qualities; however, nearly a quarter of civil servants had worked with researchers whom they considered to be poor collaborators. They noted that although the process was sometimes strained, the partnership had delivered some policy successes. These civil servants argued that pragmatism trumped personal preference and they would work with these researchers again if they believed a positive outcome was achievable. No policymakers suggested that researchers who were critical of government were necessarily poor collaborators.

### Authenticity

Six of the eight politicians thought it very important that a researcher's agenda was *“authentic”*, *“genuine”*, *“honourable”* and *“sincere”*, i.e. they were advocating on behalf of the public good *“for the right reasons”* and not because they *“liked listening to the sound of their own voice”,* or because they were attempting to secure increased funding for their program:

*Sometimes people overstep their bounds and try to overemphasise the importance of their issue when the evidence may not necessarily support it. They've got a research agenda to support, research centres to get funding for – so they put it on the agenda to get more funding from government agencies because they're salesmen of their particular illness or disease… [They] aren't making a real contribution. [They're] just tinkering at the edges.*

One minister noted: *“you don't go back to those people twice”.* Only a quarter of civil servants echoed this concern. One argued that motivation was irrelevant: *“…if they're saying the right thing, it's based on research…then I don't mind whether they're doing that out of the goodness of their heart or out of hogging the limelight. They're still doing something good”*.

### Faithful representation of research

Interviewees universally agreed that researchers' commentary or advice must be congruent with the research. Thus policymakers looked to researchers for “*honest and unbiased”, “objective”*, *“non-ideological”, “unadulterated factual quality information”* which did not *“manipulate”* the data or *“overstep”* it in any way. Politicians seemed particularly concerned to avoid researchers who gave ideologically-driven advice—*“We have enough ideology in politics”*—or who *“finessed”* the data: *“You have to have people who you can trust absolutely who will educate you, not give you spin.”*

One minister commented wryly: *“If it's going to be twisted and manipulated, leave that to me!”*

This concern about ideology also applied to institutions. Exceptions were where the politicians believed their department was partisan and wanted to balance departmental advice with a counter view. In these rare cases the politicians consulted with researchers in think tanks that were known to have a particular political leaning.

### Communication skills

Researchers' ability to communicate clearly in briefings, committees, public meetings and the media was valued by policymakers: *“they've got that skill in talking to media but then they've got that skill in talking to government and also talking to us [civil servants] without it being patronising”.* Nearly two thirds of the politicians preferred researchers who avoided dense, technical language: *“being able to understand what they are saying is actually a big, big problem”.* Therefore researchers who were *“concise”* who could *“get to the point”* and *“who are able to express complex concepts in fairly simple and easy to understand terms”* were *“the ones that really strike a chord with the policy and political people.”* Less than half the civil servants emphasised communication skills. Those who did focused on researchers' strategic communication: the ability to communicate persuasively with different audiences in different contexts. Listening and giving opinions respectfully was important. Researchers with less impressive communications skills were used for research and advice but not for persuasive or public purposes: *“if they're that kind of presenter then I won't use them. I'll use their research but I won't use them in the meeting.”*

## Discussion

Trust was at the heart of policymakers' approaches to identifying and assessing researchers. This helps to explain their preference for working repeatedly with the same tried and tested researchers, and their tendency to rely on colleagues to recommend new researchers—a recommendation being some assurance that the researcher had been informally ‘risk-assessed’.

All the attributes that policymakers' sought in their assessments of researchers could be understood using the dimensions of trustworthiness identified in the literature: *competence/ability, integrity* and *benevolence* [14], [15]. Policymakers judged researchers' competence through their: reputation, academic authority, scope of expert knowledge, applied research skills, and their understanding of government as manifested in their collaborative and communication skills. Academic credentials were seldom sufficient to convince policymakers that someone was worth listening to; an understanding and experience of pragmatic research and/or policy-shrewdness was often required to establish *“real world”* competence. Such assessments were context specific, shaped by current need, such as the type of policy development the policymaker was engaged in (e.g., developing intervention programs required different input than legislative change), and the different phases of development (e.g., exploratory discussion during agenda-setting favoured researchers with *“big ideas”* while consensus-building demanded researchers who could engage with and persuade stakeholders). Thus a researcher's reputation did not have fixed value across the repertoire of policy needs; it was considered on a fit-for-purpose basis, in much the same way that research itself is often appraised [23]–[25].

Some desirable attributes were universal. All interviewees were adamant that credible researchers must have integrity. Policymakers sought researchers who were *“fiercely independent”* and *“honest and unbiased”* and many also found it easier to trust *“honourable”* researchers who were *“authentic”* in their desire to improve public health. Yet, in the high risk politicised world of policymaking [26], the added assurance of benevolence was critical: the belief that researchers had sufficient commitment to the current policy agenda (and possibly to the policymakers themselves?) to sustain their support or, at least, withhold public opposition, through the *“ugly compromises”* of the policy process. The ideal researcher was professionally disinterested, detached from politics but dedicated to improving public health and therefore committed to their role in furthering research-informed policies.

This tension between political independence and policy commitment could pose problems. Policymakers had to trust researchers to respect confidentiality even if the researcher saw a potential to advance public health by revealing information that highlighted poor decision-making. Consequently, a researcher's media profile could be a double edged sword. Although the majority of policymakers valued and utilised researchers with media skills and contacts, some commented that this easy access to the public also made researchers a greater threat if they were critical of government. Thus *“anybody that is likely to be trouble”,* or may be *“difficult to control”*, who might *“go to a press conference and… launch an attack on the government”—*was excluded from insider conversations. While policymakers acknowledged researchers' *“democratic right”* to criticise government policy, none of them responded positively to our prompts about the role of researchers in holding governments accountable. Policymakers occasionally sought the views of individual researchers or institutions known to oppose current policy plans, sometimes in order to demonstrate openness to a breadth of advice rather than to consider it. But once policy directions had been decided policymakers seldom worked with researchers who were not *“on our side”*.

These findings may concern researchers and experts who believe that they have responsibilities to advocate for research-informed policy reform. They may be tempted to mute their advocacy to avoid the risk of becoming *persona non gratia* if they openly criticise government policy or inaction. Public health policy reform frequently calls for increased regulation, yet when anti-regulatory governments are in power many cornerstone reforms are known to be off the agenda, despite the strength of the research base. These circumstances create professional challenges both for researchers who are politically acceptable and for those who find themselves firmly “outside the policy tent” [27].

### Implications for policymakers

As our interviewees pointed out, the ways in which policymakers identify researchers would be better if they were more systematic and considered. Relying on media presence as a proxy for academic authority has self-evident risks. There are many roles for researchers that do not require them to be media-savvy, and those who are adept at media advocacy or at ‘speaking policy’ do not necessarily offer the most impartial or informed advice. These risks could be reduced through more systematic scrutiny of researchers' credibility within the wider research and policy communities. But relying wholly on interpersonal networks to identify and assess researchers is also problematic because new recruits will be more likely to share the perspective of the colleagues who recommended them. Given that there is not always a policy consensus in public health it is essential that policymakers cast their nets broadly to obtain a spectrum of advice. Knowledge brokers, NGOs, over-looked universities and research institutions, and a search of the literature (including the use of academic measures such as citation rates and/or matrices like the h-index [28]) may provide alternative sources. The work and advice of all researchers should be appraised, no matter how long-standing their connections with policy may be.

Policymakers stated that working relationships with researchers were most productive when the researchers were “*partners in the process”.* However, in order to be effective and trustworthy partners, researchers required considerable understanding of and engagement with policy. It seems that policymakers are in the best position to facilitate this: informing researchers about policy priorities, providing feedback about deliberations and outcomes, convening discussion about research requirements, and educating newer researchers about bureaucratic and government processes.

### Implications for researchers

The implications of the attributes that policymakers value in researchers need little explication: researchers who wish to influence policy will hone these attributes or not according to their goals, values, core skills and personalities. Furthermore, we suspect that our account of how researchers are assessed by policymakers will simply confirm what most policy-engaged researchers have discovered from experience. Influential public health researchers who we interviewed in an earlier study had well-developed understandings of the attributes that policymakers valued in researchers [4]: their accounts of these attributes tally well with those described by policymakers in this paper. However, there are some overarching strategic implications from our findings on the formation and influence of researcher-policymaker relationships.

These findings suggest that, in the majority of cases, the most effective method for researchers to influence policy is to work with civil servants. Civil servants were more open to being ‘cold canvassed’ than ministers and advisors; they were involved in the breadth of policy development from program improvement to legislation, at all stages of the policy cycle; and they provided briefs about researchers who approached ministers directly. Yet other relationships were also important. Policymakers' consultations with NGOs and professional bodies placed these stakeholders in a powerful position to facilitate the participation of researchers and the use of research in policy development. Similarly, positive networks with policy-engaged research colleagues helped to establish reputation with and links to policymakers. It would seem that a researcher who is well connected (and respected) across multiple policy domains significantly increases their opportunities to influence policy.

Despite our interviewees' emphasis on relationships we caution against any suggestion that researcher-policymaker networks are a panacea for the research/policy disjuncture. First, interviewees naturally focused on the aspects of policy development that they were responsible for rather than the overall trajectory of policy reform. This focus on day-to-day deliberations probably over-emphasises the role of interpersonal communication. Second, interviewees acknowledged the labyrinthine nature of the bureaucratic machine in which a multitude of individuals and interests jostle to influence policy. This prevents the smooth passage of research recommendations into policy, regardless of who champions them [29]. Researchers may be able to convince people at one strata of policy through interpersonal means, but other complementary measures are required to achieve a critical mass of support. Changes of government and high staff turnover among civil servants compound the limitations of relying on personal relationships [30]. Therefore, the other methods of contributing to policy that our interviewees identified also require attention: serving on committees, advisory groups and task forces; providing departmental and ministerial briefings; promoting research via conferences, tailored workshops, websites and newsletters; undertaking government-commissioned research; advocacy through direct lobbying; and media engagement. This last point is particularly salient given that our politician interviewees used the media as their primary means for identifying researchers.

A multifaceted approach to influencing policy allows researchers to negotiate roles and strategies in response to emerging opportunities that reflect their disciplinary skills, communicative strengths and personal preferences. Using diverse methods to influence policy also supports the process of “enlightenment” [31], that some argue is the major way in which research influences policy [32]. In this model, research-informed ideas permeate the policy process through multiple routes including family and friends, intra-organisational ‘grapevines’, and wider networks and the media, to produce a gradual conceptual change that is not easily portrayed in a direct cause and effect relationship between research findings and policy outcomes [33].

### Conclusion

This paper broadens our understanding of research-to-policy channels by describing the ways in which some research-engaged Australian state civil servants, ministers and ministerial advisors identify researchers to provide policy advice or undertake commissioned research, and how they make judgements about if and how to use these researchers.

How policymakers found and assessed researchers was strongly affected by their role. Politicians were more likely to use media presence at a proxy for researchers' merit, to express concerns about ideology, and to consider public perception of the researcher. Civil servants were more likely to canvass research and policy networks, and assess researchers on their track record using conventional academic indicators. The type of policy development the policymaker was engaged in, and the phase of development (e.g., agenda-setting, consensus building or program design) also shaped how researchers were assessed.

Assessments about researchers encompassed a range of behavioural and attitudinal attributes which clustered under the three domains of trustworthiness: *competence*, *integrity* and *benevolence*. In policy settings, researcher *competence* comprises policy pragmatism as well as scientific rigour. Viewing researcher-policymaker engagement through the lens of trustworthiness helps us to understand the tension between policymakers wanting researchers who were independent and non-ideological (attributes of *integrity*) but nonetheless *“on our side”* for the purposes of policy reform (*benevolent*).

Policymakers are encouraged to better their methods for selecting researchers and to investigate other mechanisms for identifying them. They could also maximise the many benefits of strong researcher-policymaker networks by supporting researchers in their efforts to engage more effectively with policymaking.

Researchers should adopt a multifaceted approach to policy influence. This includes cultivating responsive relationships with policymakers, community stakeholders and research colleagues, and using complementary channels of influence such as serving on committees, providing behind-the-scenes advice, diffuse dissemination of research, and education/advocacy through the media. The latter can both support and pressure policymakers to consider research in their decision-making.
